# Association of Nonalcoholic Hepatic Fibrosis with Body Composition in Female and Male Psoriasis Patients

**DOI:** 10.3390/life11080763

**Published:** 2021-07-29

**Authors:** Kinga Brunner, Péter Oláh, Mehdi Moezzi, Gabriella Pár, Áron Vincze, Zita Breitenbach, Rolland Gyulai

**Affiliations:** 1 Department of Dermatology, Venerology and Oncodermatology, Medical School, University of Pécs, Akác u. 1., 7632 Pécs, Hungary; brunner.kinga@gmail.com (K.B.); ptr.olah@gmail.com (P.O.); moezzimehdi@yahoo.com (M.M.); 2 Institute of Nutritional Science and Dietetics, Faculty of Health Sciences, University of Pécs, Vörösmarty u. 3., 7621 Pécs, Hungary; zita.breitenbach@etk.pte.hu; 3 Department of Dermatology, Medical Faculty, Heinrich Heine University, Moorenstr. 5, 40225 Düsseldorf, Germany; 4 First Department of Medicine, Medical School, University of Pécs, Ifjúság út 13., 7624 Pécs, Hungary; par.gabriella@pte.hu (G.P.); vincze.aron@pte.hu (Á.V.)

**Keywords:** psoriasis, NAFLD, hepatic fibrosis, body weight, body composition

## Abstract

Psoriasis has been associated with increased frequency of hepatic diseases. Psoriasis severity, obesity, insulin resistance, aspartate aminotransferase level, platelet count, and alcohol use are significant predictors for advanced fibrosis in psoriasis patients. Although psoriasis patients also present body composition changes (e.g., higher overall body fat, visceral fat and sarcopenia), and these have recently been reported as risk factors for hepatic fibrosis, to date, body composition has not been prospectively investigated in psoriasis in the context of liver fibrosis. In this study anthropometric assessment (body weight and body mass index (BMI)), body composition analysis (body fat%, visceral fat scores and muscle mass%), and liver stiffness measurements (using transient elastography [TE]) were done in 52 psoriasis patients undergoing methotrexate therapy. Fourteen patients (26.9%) had advanced (F3–F4) liver fibrosis. There was no correlation between the patients’ liver stiffness values and the cumulative MTX doses. On the other hand, patients with higher BMI values, total body fat% and visceral fat scores were significantly more likely to present with higher hepatic stiffness values. BMI was a significant predictor of hepatic fibrosis in both genders. In males, body fat% (R = 0.578, *p* = 0.002) and, especially, visceral fat scores (R = 0.716, *p* < 0.001) had statistically significant correlation with stiffness scores, while in females only visceral fat scores were statistically significant predictors of the liver stiffness values (R = 0.452, *p* = 0.023), and body fat% was not (R = 0.187, *p* = 0.382). Our results suggest that anthropometric data should be assessed differently in female and male psoriasis patients when evaluating liver fibrosis risk.

## 1. Introduction

Psoriasis is one of the most common chronic dermatological diseases, with an adult prevalence of 0.51 to 11.43%. Environmental and genetic factors both play a role in the development of immune-mediated cutaneous inflammation. Among the most significant environmental factors influencing psoriasis onset or disease severity/activity, are mental and physical stress [1], alcohol consumption [2], and smoking [3]. In addition, a growing body of evidence indicates that excessive body weight, nutritional factors, and diet may exacerbate the clinical manifestations of psoriasis or trigger the onset of the disease [4,5,6,7].

A significant proportion of psoriasis patients show that, in addition to skin and joint symptoms, additional comorbidities, including metabolic syndrome (MS), diabetes mellitus (DM), and cardiovascular diseases [8,9]. Hepatic diseases, such as non-alcoholic fatty liver disease (NAFLD), non-alcoholic steatohepatitis (NASH) and, in severe cases, hepatic fibrosis, have also been linked to psoriasis. The prevalence of NAFLD is as high as 45–47% among psoriasis patients, while NASH affects one in five patients [10,11]. Moreover, according to recently published data, more than 14% of severe psoriasis patients have liver fibrosis [12]. While liver biopsy has traditionally been the gold standard for monitoring methotrexate-induced liver fibrosis in psoriasis patients, non-invasive procedures, such as transient elastography [13,14,15], shear-wave elastography [16] or laboratory methods [17] has been shown, more recently, to provide comparable accuracy.

Although historically methotrexate (MTX), one of the most used systemic antipsoriatic agents, has been linked with hepatotoxicity in psoriasis, more recent data suggests that metabolic syndrome (obesity, insulin resistance, hyperglycemia, and dyslipidemia) is the leading cause of NAFLD and fibrosis both in the general population and in psoriasis patients [18]. Although body composition differences, specifically, increased fat percentage has recently been reported as an independent risk factor for incident NAFLD [19], to date body composition has not been prospectively investigated in psoriasis in the context of liver fibrosis. Therefore, we undertook a study to assess the association of liver fibrosis with body composition factors in a population of severe psoriasis patients.

## 2. Materials and Methods

This study was approved by the Regional Research Ethical Committee of the University of Pécs (ethical permission file number: 7947-PTE 2019) and was conducted under the Declaration of Helsinki. Informed consent was obtained from all subjects involved in the study.

### 2.1. Patients

**Figure 1:**
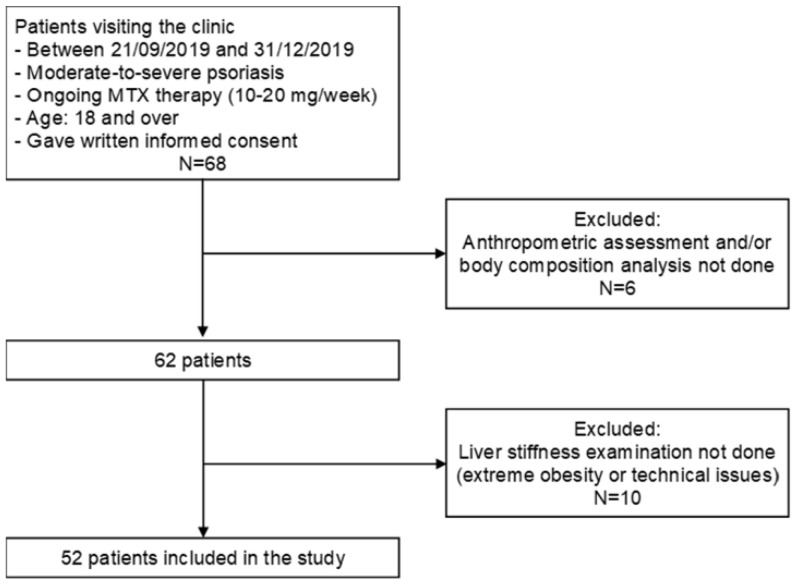
Flow chart of patient inclusion and exclusion in the study.

Consecutive psoriasis patients (n = 68, male = 36, female = 32) ≥18 years old undergoing MTX therapy at the Psoriasis Outpatient Unit of the Department of Dermatology, Venereology and Oncodermatology, Medical School, University of Pécs, were recruited prospectively between 21 September, 2019 and 31 December, 2019 (Figure 1). All patients had moderate-to-severe or severe psoriasis, and most patients were treated with 10–20 mg MTX once weekly with 4–12 mg/week folic acid supplementation. Moderate-to-severe psoriasis was defined as psoriasis area and severity index (PASI) ≥ 10 and/or dermatology life quality index (DLQI) ≥ 10.

Demographic data and cumulative MTX doses were extracted from the electronic medical records of the patients. In addition, physical activity levels and the smoking habits of the participants were assessed at study entry using a questionnaire.

### 2.2. Anthropometric Assessment and Body Composition Analysis

Body weight was measured without shoes in light clothing, 2 h after eating. BMI was calculated as the weight divided by the height squared (kg/m^2^). WHO recommended categories were used to stratify patients according to BMI: normal (18.5–24.9 kg/m^2^), pre-obesity (25–29.9 kg/m^2^), obesity class I (30–34.9 kg/m^2^), obesity class II (35–39.9 kg/m^2^), obesity class III (>40 kg/m^2^) [20].

Body composition was assessed using a bioelectrical impedance analyzer (single-frequency BIA—bioelectrical impedance analysis Omron BF511). Values of body fat percentages obtained were adjusted to gender and age and categorized into low, normal, high, and very high groups, using the criteria of Gallagher et al. for white population [21].

Visceral fat categories were the following: normal (0–9), high (10–15), very high (>15). In 6 patients, anthropometric assessment and/or body composition analysis was not possible due to technical issues—these patients were excluded from the analysis.

### 2.3. Liver Fibrosis Evaluation

Liver stiffness measurements were performed using FibroScan 502 (Echosens, Paris, France). Patients were examined with the M-probe. Liver stiffness was measured in kPa and stiffness values were converted into fibrosis scores as follows: F0–F1–no fibrosis-mild fibrosis (≤8.2 kPa); F2–moderate fibrosis (>8.2 to 9.7 kPa); F3–severe fibrosis (>9.7 to 13.6 kPa); F4–cirrhosis (>13.6 kPa) [22]. Patients with increased liver fibrosis scores (F2–F4) were advised to stop methotrexate therapy and were evaluated for switching to other systemic/biologic antipsoriatic therapy. In 10 patients, liver stiffness data could not be obtained due to extreme obesity or other technical issues and, thus, they were excluded from the analysis.

### 2.4. Statistical Analysis

Descriptive statistics (mean, standard deviation, frequency) were analyzed using SPSS v24. Comparative statistical analyses and visualization were carried out using R v3.5.1 and Numbers v3.6.2. Following normality tests, chi-square, paired and unpaired *t*-test, ANOVA, Kruskal–Wallis test, and linear regression were applied as appropriate. Gender-based comparisons (Table 1) were carried out using Student’s *t*-test and Mann–Whitney test for individual continuous or ordinal variables (e.g., mean body weight) and chi-square test was applied to categorical variables (e.g., BMI category). Contrasts among multiple groups (e.g., Table 2) were determined using ANOVA and Kruskal–Wallis tests. For the correlation of pairs of continuous variables, liner regression was carried out and Pearson’s correlation coefficients determined (Figures 3 and 4). The exact statistical tests applied are indicated in the respective figure legends. Significance criterion of *p* < 0.05 was applied.

## 3. Results

### 3.1. Patient Demographics, Anthropometric Assessment, and Body Composition Analysis

Overall, 52 patients had complete anthropometric assessment, body composition analysis and liver fibrosis evaluation, and were included in the subsequent analysis (Table 1). Mean age was 54.0 years and was not statistically different between females and males (mean age females 52.9 ± 13.2, males 55.2 ± 13.6 years). Patients categorized their physical activity level as follows: very good (n = 6, 11.5%), good (n = 12, 23.1%), average (n = 22, 43.3%), bad (n = 12, 23.1%), very bad (n = 0). Smoking habits of the patients were the following: never smoked (n = 17, 32.7%), quit smoking (n = 21, 40.4%), smokes < 5 cigarettes per day (n = 4, 7.7%), smokes 5–10 cigarettes per day (n = 5, 9.6%), smokes 11–20 cigarettes per day (n = 4, 7.7%), smokes > 20 cigarettes per day (n = 1, 1.9%).

All patients had moderate-to-severe psoriasis and were treated at study entry with methotrexate. Median MTX treatment duration time was 4.8 ± 3.0 years (interquartile range, 0–13 years), mean weekly methotrexate dose was 14.2 ± 2.6 mg (interquartile range, 5–20 mg) and mean cumulative MTX dose was 2273.8 ± 2238.7 mg (Table 1). Statistically, there was no difference between males and females in terms of the average weekly and cumulative MTX doses and the mean treatment duration times.

Most patients, both males and females, were overweight: mean body weight was 91.6 ± 17.7 kg, and mean BMI 31.4 ± 5.1 kg/m^2^ in the total population. Male patients weighed significantly more (96.7 ± 17.0 kg) than females (86.4 ± 17.1 kg; *p* = 0.034), however, mean BMI was almost identical in males and females (31.1 ± 4.5 kg/m^2^ and 31.7 ± 5.7 kg/m^2^, respectively; *p* = 0.664). Of the 52 patients only 4 had normal BMI (Table 2). Nineteen patients were pre-obesity category (BMI 25 to <30 kg/m^2^) and 29 were obese (BMI ≥30 kg/m^2^). Of the obese patients 17 fell into obesity class I (BMI 30 to <35 kg/m^2^), 10 into obesity class II (BMI 35 to <40 kg/m^2^), and 2 into obesity class III (BMI ≥40 kg/m^2^) category. Mean body fat% was 36.3 ± 8.8 in the total population (Table 2). Compared to men, women had significantly higher body fat% (females 41.2 ± 7.6, males 31.3 ± 7.1, *p* < 0.001); however, patients with age and gender adjusted normal, high and very high body fat percentage values were similar in both males and females (3, 8, and 15, and 3, 11, and 12, respectively). Even 3 of the 4 patients with normal BMI had higher than normal body fat percentage values. Women also had significantly lower visceral fat scores (females 10.2 ± 3.2, males 16.3 ± 5.5, *p* < 0.001), and muscle mass% values (females 25.6 ± 5.2, males 30.3 ± 5.0, *p* = 0.002) than males. In the total population patients in the higher BMI categories were significantly more likely to have higher body fat% and visceral fat scores, and lower muscle mass% values (*p* < 0.001, *p* < 0.001 and *p* = 0.008, respectively). Both in female and male patients, body fat% and visceral fat scores were significantly higher in patients with increasing BMI values. Muscle mass% values were significantly lower in case of male patient in higher BMI groups, while in females, a negative but not significant trend was seen. As expected, mean muscle mass% values were higher in males than in females in all groups stratified according to BMI. Compared to males, mean body fat% values were higher in females in all BMI groups, however, despite lower overall body fat% mean visceral fat scores were higher in males than in females.

### 3.2. Liver Fibrosis Evaluation

**Figure 2:**
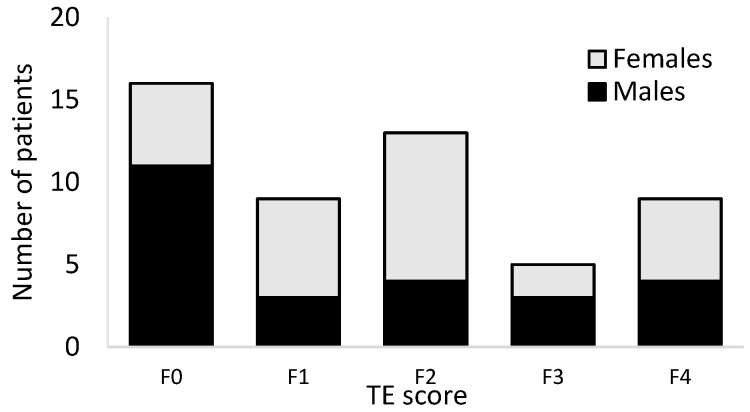
Number of female and male patients with different TE scores. No statistical difference between females and males were detected (ANOVA, *p* = 0.247), TE (transient elastography).

Based on the liver stiffness measurements, 16 patients had no fibrosis (transient elastography [TE]) score F0) (Figure 2). Mild (F1), moderate (F2), and severe (F3) fibrosis was detected in 9, 13 and 5 patients, respectively. Nine patients had liver stiffness values corresponding to cirrhosis (F4). Of the 16 patients with F0 score 11 were males and 5 were females. The total number of patients with advanced fibrosis (F3 + F4) were equal in females and males (2 + 5 and 3 + 4, respectively), with no statistical difference between females and males.

### 3.3. Correlation of Liver Fibrosis and Cumulative Methotrexate Dose, Body Mass Index and Body Composition Values

**Figure 3:**
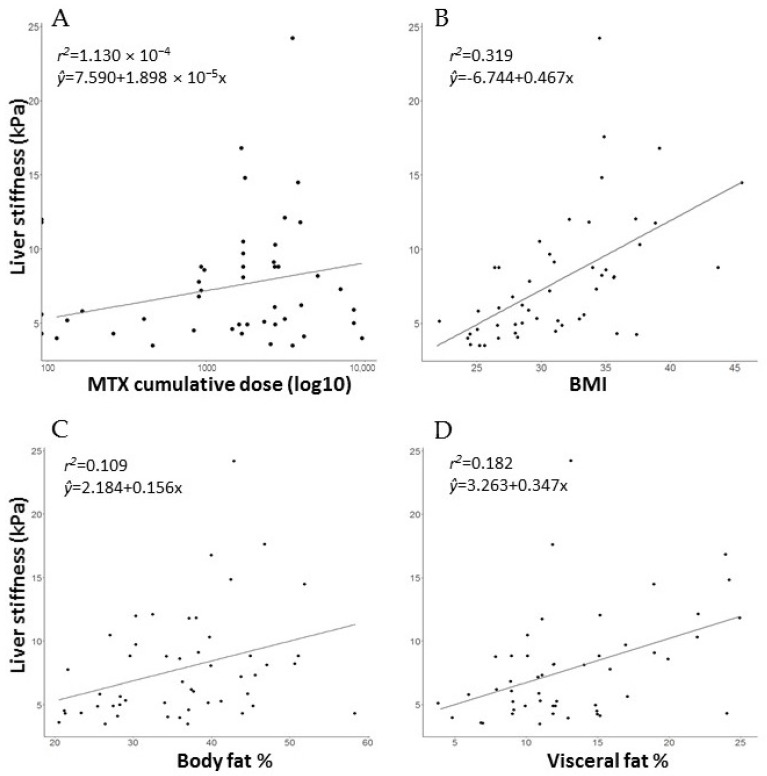
Correlation of liver stiffness values (kPa) with (**A**) cumulative methotrexate doses, (**B**) BMI (body mass index) and (**C**) total body fat% values, and (**D**) visceral fat scores in psoriasis patients. Each dot represents a patient. Dots may be slightly jittered to avoid overlaps. Solid lines represent the lines of best fit. No correlation between the cumulative methotrexate dose and the liver stiffness values was observed (*p* = 0.803), while BMI, total body fat% and visceral fat scores showed strong correlation with the stiffness values (*p* < 0.001, *p* = 0.016 and *p* = 0.001, respectively).

Since all patients received methotrexate, and methotrexate has been historically associated with liver toxicity, we next assessed whether patients exposed to higher MTX doses were more likely to have more severe fibrosis scores. There was no correlation between the patients’ liver stiffness values and the cumulative MTX doses (r^2^ = 1.130× 10^−4^, *p* = 0.803) (Figure 3A). On the other hand, patients with higher BMI values, total body fat% and visceral fat scores were significantly more likely to present with higher liver stiffness values (Figure 3C,D). Simple linear regression between liver stiffness values and BMI, body fat% and visceral fat scores showed significant positive correlation in the total population (Table 3). Moreover, patients in the total population stratified to higher BMI categories (pre-obesity, obesity class I or class II + III) were statistically significantly more likely to have more severe stiffness values (*p* = 0.001) (Table 2).

### 3.4. Gender-Specific Differences in the Association of Liver Fibrosis Scores with Body Mass Index and Body Composition Values

**Figure 4:**
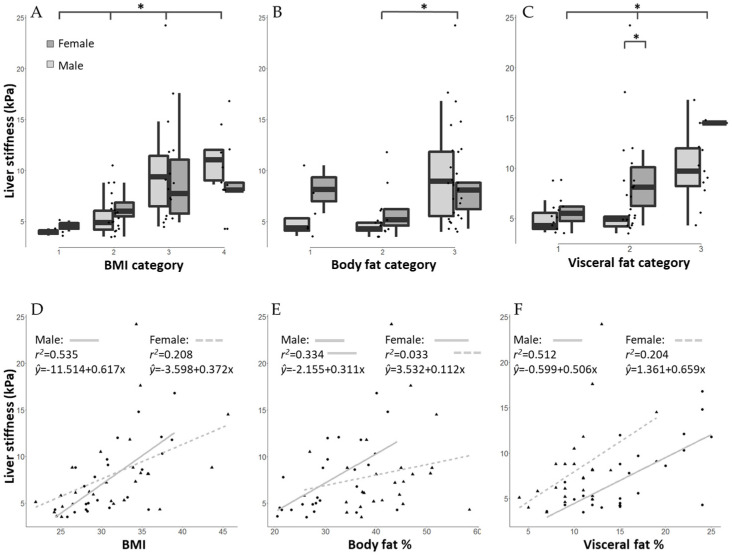
Mean liver stiffness values (kPa) of psoriasis patients in different BMI—body mass index (**A**), body fat% (**B**), and visceral fat score (**C**) categories. Females are represented as dark grey bars, males as light grey bars. (**A**) BMI categories: 1. Normal (BMI 18.5 to <25 kg/m^2^); 2. Pre-obesity (25 to <30 kg/m^2^); 3. Obesity class I (30 to <35 kg/m^2^); 4. Obesity class II-III (≥35 kg/m^2^). Significant differences were found in the liver stiffness values across BMI categories (Kruskal–Wallis and Dunn’s post-hoc test, *p* < 0.05). No significant difference was found by gender within BMI categories. B) Body fat% categories. Values of body fat percentages obtained were adjusted to gender and age and categorized into low (no patient, not shown), normal (1), high (2), and very high (3) groups. Significant differences were found in the liver stiffness values between body fat% categories 2 and 3 (Kruskal–Wallis and Dunn’s post-hoc test, *p* = 0.025). C) Visceral fat score categories. 1. Normal (0–9); 2. High (10–15); 3. Very high (>15). Asterisks (*) indicate differences with *p* < 0.05. Significant differences were found in the liver stiffness values across visceral fat categories (Kruskal–Wallis and Dunn’s post-hoc test, *p* < 0.05). Significant difference was found by gender within visceral fat category 2 (*p* = 0.029). Correlation of liver stiffness values of female and male psoriasis patients with BMI (**D**), body fat% (**E**), and visceral fat% (**F**). Each triangle represents a female patient, and each dot a male patient. Marks may be slightly jittered to avoid overlaps. Solid and dotted lines represent the lines of best fit for male and female patients, respectively.

In both males and females, statistically significant correlation was found between the BMI and the stiffness values (*p* < 0.001 and *p* = 0.002, respectively) (Table 3, Figure 4A,D). There was no significant difference within BMI categories between genders (Figure 4A,D). In males body fat% and visceral fat scores showed statistically significant correlation with hepatic stiffness values (*p* = 0.002 and *p* < 0.001, respectively) (Table 3, and Figure 4B,C (light grey bars) and Figure 4E,F (solid lines)). On the other hand, in females body fat% was not significant predictor of liver stiffness (*p* = 0.382) (Table 3, Figure 4B [dark grey bars] and Figure 4E (dotted lines)), while visceral fat scores showed statistically significant correlation with hepatic stiffness values (*p* = 0.023) (Table 3, Figure 4C [dark grey bars] and Figure 4F (dotted lines)).

## 4. Discussion

In this cohort of severe psoriasis patients taking methotrexate, we assessed whether cumulative MTX dose or anthropometric data influence the development of liver fibrosis. Most patients in our study cohort were pre-obese or obese, with increased BMI, body fat percentage, and visceral fat scores. According to BMI, 19 of the 52 patients (36.5%) were pre-obese and 29 (55.8%) were obese. While body mass index (BMI) is the most commonly used measure to monitor the level of obesity, it is not suitable to assess body composition differences, especially concerning fat distribution. This is particularly notable, as patients with psoriasis also present specific body composition changes, including higher overall body fat, visceral fat, and sarcopenia [23]. A recent study also found that, despite normal BMI values, 22.5% of men and 5.5% of women with psoriasis had increased fat mass [24]. Our results confirmed these findings, as we have also found that most of the patients in our cohort—even 3 of the 4 patients with normal BMI—had higher than normal body fat percentages.

In addition to an overall increased frequency of obesity in psoriasis, body composition of psoriatic men seems to differ from that of women. According to a recent study, female psoriatic patients have significantly higher fat mass and adipose tissue percentages [24]. Our results are in part consistent with these results, as we have also found that women had significantly higher body fat%, lower visceral fat scores and muscle mass% values. Using age and gender adjusted body fat% categories, however, we have not found significant differences between female and male patients.

In a recent publication, 77 of 333 (14.1%) severe psoriasis patients had advanced liver fibrosis as diagnosed by transient elastography [12]. Interestingly, in our cohort more than 25% of patients presented with advanced (F3–F4) liver fibrosis. Several factors, including psoriasis disease severity, previous and concomitant therapies, lifestyle, nutrition, smoking habits could have contributed to the relatively high frequency of liver disease in our patient cohort. As methotrexate has been historically linked with hepatotoxicity in psoriasis, and in our cohort all patients received MTX therapy, MTX seemed a likely culprit for the high frequency of liver fibrosis. Several recent publications, however, found no association between cumulative MTX doses and liver fibrosis in psoriasis [25,26]. Our findings confirm these results and support the hypothesis that MTX *per se* is not a significant risk factor for liver fibrosis in psoriasis. It must be emphasized, however, that many psoriasis patients suffer from comorbid conditions (such as obesity) that can present significant baseline risk for liver fibrosis, in which case MTX treatment may constitute additional hazard. As our patients were otherwise asymptomatic and had normal liver function test results, our results also underline the importance of liver fibrosis screening and the value of transient elastography in the follow-up of psoriasis patients.

In contrast to cumulative MTX doses, higher BMI values, total body fat% and visceral fat scores were significantly associated with higher liver fibrosis values in our patient population. NAFLD and consequent fibrosis development has been strongly linked with obesity [27]. People with psoriasis have high rates of obesity [28], which partly explains the increased incidence of NAFLD and fibrosis in the psoriatic patient population. Most likely for this association is that psoriasis is a systemic inflammatory disease and that the systemic pathophysiological mechanisms involved in the development of skin symptoms may also lead to pathological changes in other organs, such as the liver. In a multivariate model, a combination of psoriasis severity, central obesity, insulin resistance, aspartate aminotransferase level, platelet count, and alcohol use were the most significant predictors for advanced liver fibrosis in psoriasis patients [12].

Moreover, female sex has recently been found to be a significant independent risk factor for the development of hepatic fibrosis in psoriasis patients [29]. Interestingly, while BMI was a significant predictor of hepatic fibrosis in both genders, and in males both body fat% and visceral fat scores had statistically significant correlation with stiffness scores, in females only visceral fat scores were statistically significant predictors of the liver stiffness values, and body fat% was not. On the other hand, female patients with high visceral fat ratio (10–15%) compared to male patients in the same visceral fat category were significantly more likely to present with elevated liver stiffness values. Recently, lower skeletal muscle mass combined with abdominal obesity has been shown to be strongly associated with the presence of NASH only in men, and not in women, further emphasizing that body composition values may need different interpretation in females and males. Whether these differences can be attributed to different endogenous (e.g., genetic, hormonal, etc.) or exogenous (e.g., nutritional, lifestyle, etc.) factors, remains a matter of debate.

We are aware that there are several limitations to this study. The number of participants in the study is lower than optimal, as the recruitment of patients into the study had to be stopped due to the COVID-19 outbreak in January 2020. While we have also collected information about the diet, smoking habits and physical activity levels of the participants, the limited sample size did not allow for adjusting for the potential bias caused by these factors likely affecting psoriasis, NAFLD, and body composition. In addition, extreme obese patients had to be excluded from the investigations, because transient elastography could not be performed in this population. Inclusion of this population, however, would have likely resulted in the detection of more frequent and more severe hepatic fibrosis. Moreover, controlled attenuated parameters of transient elastography, reflecting steatosis, were not accumulated in our patient population, and thus, we could not assess the association of the patients’ visceral fat content and liver steatosis. Finally, our bioimpedance analysis equipment only provides calculated body fat percentages and visceral fat levels without raw bioelectrical values, limiting more detailed analysis.

In conclusion, our results present gender specific differences in psoriasis patients regarding BMI and body composition (total body fat and visceral fat) values, in association with liver fibrosis development, and warrant further investigations to clarify the pathogenetic background and the clinical significance of these findings.

## Figures and Tables

**Table 1:** Demographic data of patients (n = 52).

	Total n = 52	Males n = 26	Females n = 26	*p* Value *
Age (years)	54.0 ± 13.4	55.2 ± 13.6	52.9 ± 13.2	0.537
Body weight (kg)	91.6 ± 17.7	96.7 ±17.0	86.4 ± 17.1	0.034
BMI (kg/m^2^)	31.4 ± 5.1	31.1 ± 4.5	31.7 ± 5.7	0.664
Body fat (%)	36.3 ± 8.8	31.3 ± 7.1	41.2 ± 7.6	0.001
Body fat categories				
Low	0	0	0	0.668
Normal	6	3	3	
High	19	8	11	
Very high	27	15	12	
Weekly MTX dose (mg)	14.2 ± 2.6	14.9 ± 1.9	13.5 ± 3.0	0.071
Duration of MTX treatment (years)	4.8 ± 3.0	4.5 ± 2.7	4.9 ± 3.3	0.672
Cumulative MTX dose (mg)	2273.8 ± 2238.7	2284.8 ± 2345.0	2262.3 ± 2171.0	0.972

* *p* values refer to Student’s *t*-test and Mann–Whitney test results between male and female for data in single rows. For body fat category, *p*-value refers to the chi-square test.

**Table 2:** Body composition and liver stiffness values (kPa) in the total patient population and in patient subgroups stratified according to BMI (body mass index) values.

BMI Category		Total (n = 52)	Normal (n = 4)	Pre-Obesity (n = 19)	Obese I (n = 17)	Obese II + III (n = 12)	*p* Value *
Body fat (%)	Total	36.3 ± 8.8	27.6 ± 7.6	31.1 ± 6.2	39.2 ± 7.7	43.2 ± 7.6	<0.001
	Male	31.3 ± 7.1	20.9 ± 0.6	28.3 ± 4.5	31.8 ± 7.5	38.6 ± 3.8	0.001
	Female	41.2 ± 7.6	34.1 ± 0.1	35.1 ± 6.1	43.2 ± 4.2	49.7 ± 6.7	<0.001
Visceral fat score	Total	13.3 ± 5.4	6.3 ± 2.2	10.9 ± 2.9	13.3 ± 4.1	19.3 ± 5.5	<0.001
	Male	16.3 ± 5.5	8.0 ± 1.4	12.6 ± 2.3	17.8 ± 3.4	23.1 ± 1.9	<0.001
	Female	10.2 ± 3.2	4.5 ± 0.7	8.5 ± 1.6	10.9 ± 1.7	13.8 ± 3.7	<0.001
Muscle mass (%)	Total	28.0 ± 5.5	32.7 ± 6.0	30.1 ± 5.9	26.7 ± 4.5	24.6 ± 3.8	0.008
	Male	30.3 ± 4.9	37.8 ± 1.2	31.9 ± 3.5	28.9 ± 6.7	26.7 ± 2.1	0.015
	Female	25.6 ± 5.1	27.5 ± 1.5	27.7 ± 7.8	25.4 ± 2.2	21.7 ± 3.9	0.209
Liver stiffness value (kPa)	Total	7.8 ± 4.2	4.3 ± 0.6	5.8 ± 2.0	9.8 ± 5.3	9.8 ± 3.9	0.001
	Male	7.5 ± 3.8	4.0 ± 0.5	5.4 ± 1.7	9.3 ± 3.9	10.7 ± 4.1	0.010
	Female	8.2 ± 4.7	4.6 ± 0.8	6.4 ± 2.2	10.1 ± 6.3	8.8 ± 3.7	0.159

* *p* values refer to ANOVA and Kruskal–Wallis statistical test results between BMI categories (Normal-Obese II + III) mean ± SD.

**Table 3:** Linear regression between liver stiffness values (kPa) and BMI, body fat% and visceral fat score.

		R	r^2^	B	CI	*p* Value
BMI	Total	0.565	0.319	0.467	0.269–0.665	<0.001
	Male	0.731	0.535	0.617	0.369–0.865	<0.001
	Female	0.456	0.208	0.311	0.122–0.501	0.002
Body fat%	Total	0.331	0.110	0.156	0.027–0.285	0.019
	Male	0.578	0.334	0.311	0.122–0.501	0.002
	Female	0.187	0.033	0.112	−0.149–0.374	0.382
Visceral fat score	Total	0.427	0.182	0.347	0.134–0.561	0.002
	Male	0.716	0.512	0.506	0.293–0.719	<0.001
	Female	0.452	0.204	0.659	0.099–1.220	0.023

*p* < 0.05 was considered statistically significant. BMI: body mass index; R: correlation coefficient; r^2^: determination coefficient; B: regression coefficient; CI: confidence interval.

## Data Availability

This study did not report any data.
